# A Pelvic Abscess and a Pelvic Stone Secondary to a Ureteral Calculus

**DOI:** 10.1155/2024/2382520

**Published:** 2024-09-04

**Authors:** Bo-Ran An, Ze-Peng Ma, Chao Gao

**Affiliations:** ^1^ Gastroenterology Department Affiliated Hospital of Hebei University, No. 212 Yuhua East Road, Baoding, Hebei Province 071000, China; ^2^ Department of Radiology Affiliated Hospital of Hebei University, No. 212 Yuhua East Road, Baoding, Hebei Province 071000, China; ^3^ 2nd Ward Department of Urology Affiliated Hospital of Hebei University, No. 212 Yuhua East Road, Baoding, Hebei Province 071000, China

**Keywords:** pelvic abscess, pelvic stone, ureteral calculus

## Abstract

The patient presented with abdominal pain for the first time 10 years ago and was diagnosed with a left ureteral calculus, left hydronephrosis, and hydroureter. The patient's abdominal pain disappeared after palliative treatment, but he refused any treatment measures for his calculus and hydrops. He was readmitted due to chronic pelvic pain 8 years ago and was diagnosed with a pelvic abscess and left renal atrophy after imaging examination. We performed pus aspiration treatment under the guidance of transrectal B-mode ultrasound and used antibiotic fluid for purulent cavity rinse, followed by intravenous injection of antibiotics. The abscess shrank in follow-up magnetic resonance imaging (MRI), and the pain symptom disappeared in his pelvic. We followed up with the patient for 6 months, and he had no symptoms related to his pelvic abscess that was diagnosed before. Recent abdominal computed tomography (CT) images revealed that his left kidney atrophy still exists, and a pelvic stone was found at the site of the original abscess. This case once again proves that a ureteral calculus should be treated in time; otherwise, it can lead to serious complications such as a pelvic abscess and renal atrophy. A pelvic stone can be caused by a ureteral calculus migration. Minimally invasive treatments have minimal damage to the body and are widely applicable, and the patient was cured by one of them, abscess aspiration, which implies that they can also be used for patients who cannot tolerate surgical procedures.

## 1. Introduction

Ureteral calculi are a common disease in urology, and imaging examination is the main method for diagnosing a ureteral calculus. Conventional surgical procedures are no longer the main method for treating ureteral calculi. Currently, minimally invasive treatment has become the main method for treating ureteral calculi, and most patients can recover.

A ureteral calculus can often cause hydronephrosis and hydroureteral. Long-term ureteral obstruction often causes nephron atrophy on the obstructed side [1], and this patient also experienced renal atrophy. Due to the functional compensatory effect of the healthy kidney to that of the obstructed side, unilateral renal atrophy often does not lead to azotemia. Bilateral ureteral obstruction can lead to obstructive renal insufficiency. After ureteral obstruction occurs, it can cause perirenal edema [2]. If the obstruction is not treated in time, it can often lead to local infections, such as urinary tract infection [3] and perirenal abscess [4]. Some patients may develop intra-abdominal abscesses [5, 6]. It may also develop a pelvic abscess, as in this case.

The pelvic abscess and the pelvic stone that occurred in this patient are considered to be caused by a ureteral calculus, both of which migrated to the pelvic cavity.

## 2. Case Presentation

**Figure 1:**
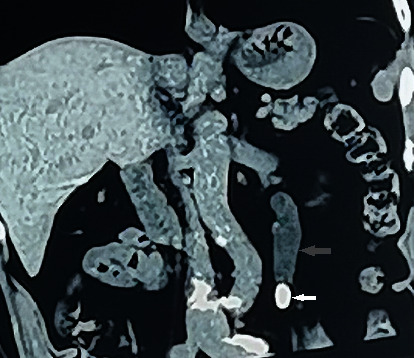
CT image in 2013 shows left ureteral effusion (grey arrow), with calculus completely obstructing the ureter (white arrow; the low-density shadow around the calculus is considered as ureteral wall edema caused by the calculus).

**Figure 2:**
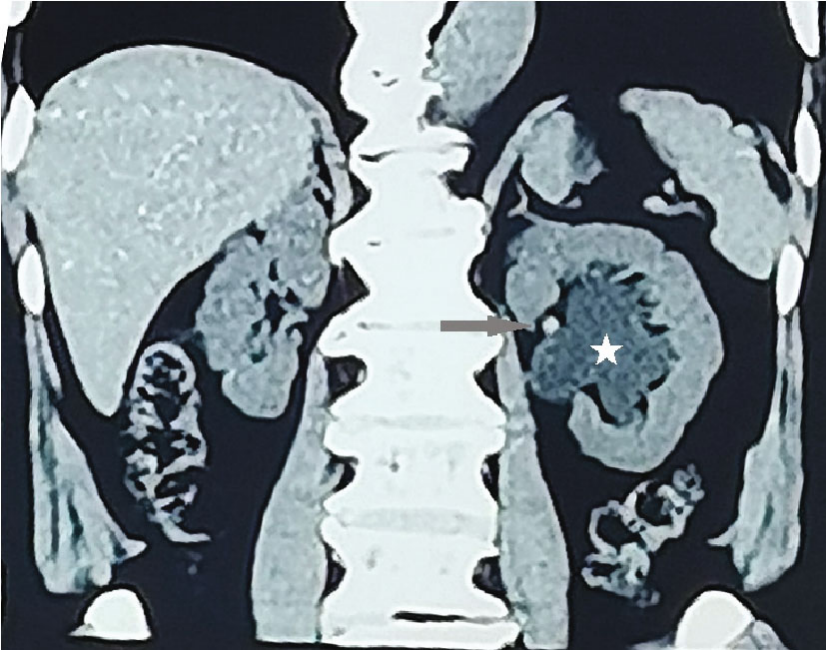
CT image in 2013 shows left renal pelvis effusion (white star) with a visible small stone shadow (gray arrow).

**Figure 3:**
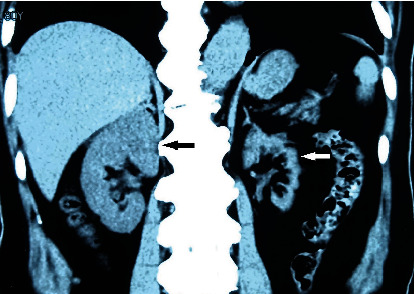
CT image in 2015 shows left renal atrophy, renal cortex thinning (white arrow), and right kidney compensatory hypertrophy (black arrow).

**Figure 4:**
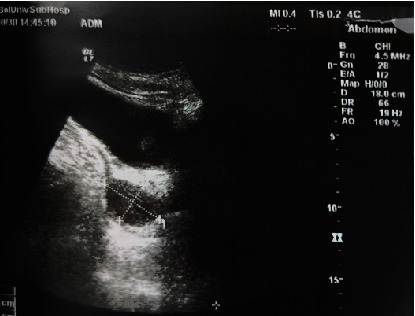
B-mode ultrasound in 2015 revealed a fluid sonograph shadow in the pelvic, posterior to the bladder.

**Figure 5:**
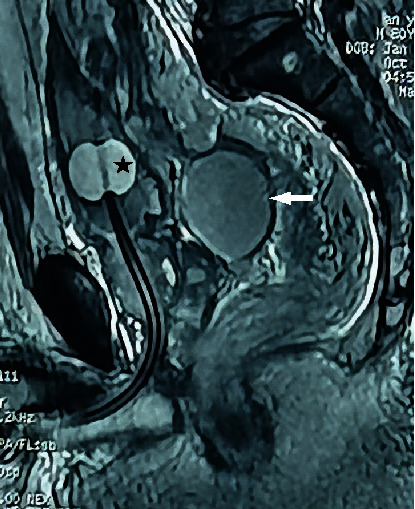
A mixed signal shadow can be seen as a quasi elliptical on MR T2WI (white arrow), located between the rectum and bladder. The left high signal shadow (hollow in middle, black star) is the water bag of the Foley catheter, located in the bladder at the internal urethral orifice.

**Figure 6:**
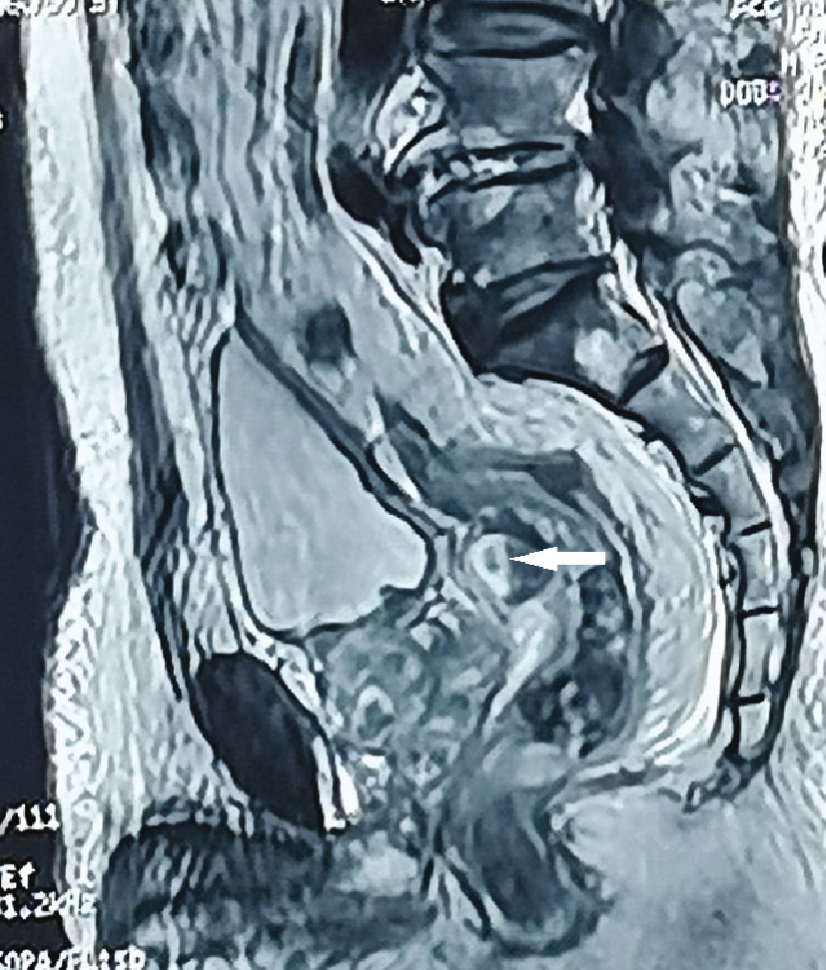
Pelvic abscess basically disappears on follow-up MRI (white arrow) after aspiration in 2015.

**Figure 7:**
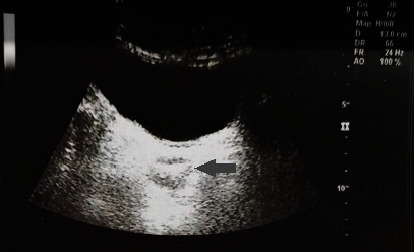
The follow-up B-mode ultrasound in 2015 revealed mixed sonograph shadow, with hyperechoic in its center, posterior to the bladder.

**Figure 8:**
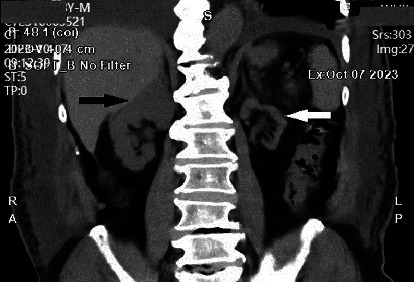
CT image in 2023 shows left renal atrophy not recovered (white arrow). Right kidney compensatory hypertrophy (black arrow).

**Figure 9:**
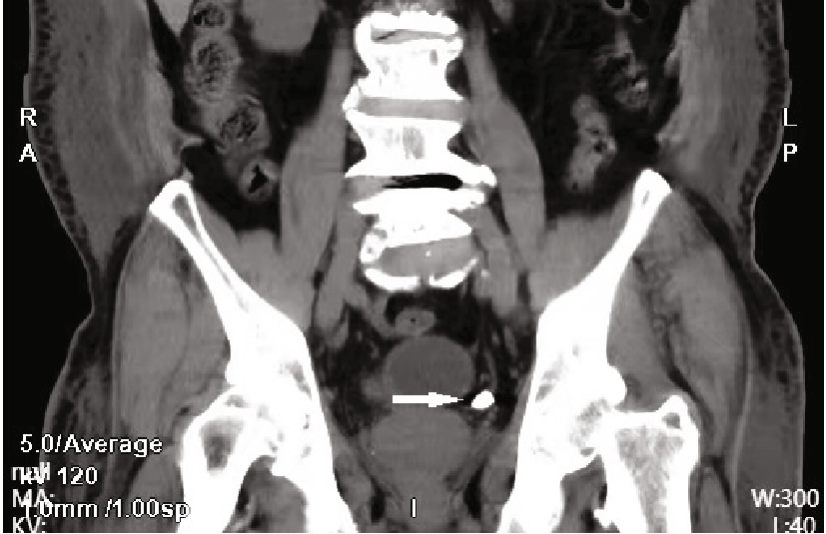
CT image on coronal view in 2023 shows a pelvic stone near the bladder (white arrow).

**Figure 10:**
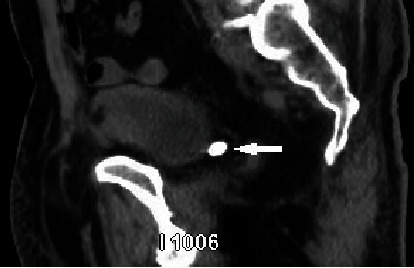
CT image on sagittal view in 2023 shows a pelvic stone posterior to the bladder (white arrow).

An 88-year-old patient was admitted to our hospital several times. In 2013, he underwent a CT scan for his abdominal pain, which revealed a left ureteral calculus, left ureteral, and left renal pelvis hydronephrosis (Figures 1 and 2). The laboratory assay showed normal renal function. So, he was diagnosed with a ureteral calculus and other related diseases for the first time. The patient said he had a history of hypertension and atrial fibrillation for many years. There are many minimally invasive treatment methods for treating urolithiasis, and we explained to him that they were safe in most cases, but he still refused to accept any surgical intervention. So, we gave him palliative treatment, and his abdominal pain disappeared after that, but the stone remained in the ureter without treatment. When we investigated his routine history in 2015, he said he had recurrent low fever after his discharge, and it can be relieved by antimicrobial agents such as norfloxacin. The patient also said he had experienced a high fever (39°C or more) accompanied by abdominal pain in late 2014. The local general practitioner considered the presence of infection in his abdomen and applied antimicrobial agents to him for treatment. After his high fever subsides, the patient often felt pain in his pelvic or hypogastrium and recurrent low fever, and the symptoms can also be relieved by being administered antimicrobial agents. He was admitted in 2015 for treatment of his pelvic pain and the lower fever. His left kidney had atrophied and a thinned cortex that was shown on CT images (Figure 3). B-mode ultrasound revealed a fluid sonograph shadow in the pelvic cavity (Figure 4). T2-weighted images of MR appeared to contain mixed signal shadows in his pelvic cavity (Figure 5). Therefore, a pelvic abscess was diagnosed after examination. The pelvic abscess was speculated to have originated from the ureteral calculus. After the diagnosis was established 3 days, the patient was subjected to a transperineal abscess puncture guided by transrectal B-mode ultrasound, which was successful and allowed approximately 40 mL of pus to be extracted by aspiration. The extracted pus was sent for routine culture both anaerobic and aerobic. Subsequently, the purulent cavity was flushed by a solution of levofloxacin, because one kind of fluoroquinolone was effective in alleviated his abdominal pain before admitted, and ornidazole solution, because most flora within intra-abdominal abscess originated from the intestine which often comprise anaerobic bacteria. A solution containing two kinds of antibiotics was injected into the abscess cavity for retention after the flush. The patient was also treated intravenously with these antibiotics after aspiration. The follow-up MRI and the B-mode ultrasound after 3 days of treatment revealed that the shadow clearly contracted (Figures 6 and 7), but mixed signal shadow remained in the MRI images, and a hyperechoic shadow was shown in its center in B-mode ultrasound, which implied that the original abscess significantly shrank. His abdominal pain and fever disappeared before discharge. Additionally, postoperative pus culture revealed the presence of anaerobic bacteria and *Escherichia coli*. Therefore, antibiotics were selected to treat the aforementioned bacterial infection and administered for a further 3 weeks. We followed up with the patient for 6 months, and he reported no pelvic discomfort. He recently returned to our hospital for other health problems. He underwent abdominal and pelvic CT scans (Figures 8, 9, and 10) upon our request. These images show that his left kidney remained atrophy and that a stone was present in the location of the original pelvic abscess, beyond the pelvic organs. Now the patient has died because of his serious illness, leaving the pelvic stone untreated.

## 3. Discussion

This case lasted for one decade. The patient was diagnosed with a ureteral calculus when he first admitted in 2013, which was accompanied by hydroureter and hydronephrosis. The laboratory assay showed that the renal function was normal in 2013, which was due to the compensatory effect of the contralateral kidney. Subsequent CT scans confirmed compensatory hypertrophy of the normal side of the kidney. If the kidney in a nonureteral calculus side had developed lesions before, it may lead to renal dysfunction [7]. It can develop into severe urinary tract infections when ureteral obstruction is caused by a ureteral calculus [3]. The recurrent low fever that the patient experienced in 2014 indicated the occurrence of infection caused by the ureteral obstruction. He had not received treatment in time when the infection occurred due to the lack of follow-up. The recurrent fever and abdominal pain the patient experienced in 2014 and 2015 were the evidence of the existence of infections, which might be in the abdominal cavity or in the pelvic cavity because pain in the abdomen or pelvic accompanied by fever is a characteristic symptom of peritoneal or pelvic cavity infections. The images the patient had been examined did not show a left ureteral calculus, hydroureter, and hydronephrosis when he was admitted in 2015, so we speculate that complete obstruction of the ureter caused by a ureteral calculus led to hydroureter and hydronephrosis, which was followed by purulent nephropathy, and ultimately leading to necrosis of the ureteral wall. The images showed renal atrophy, which was caused by high pressure in the renal pelvis. There are many reports in literature that high pressure in the renal pelvis can lead to nephron atrophy [1]. The necrosis of the ureteral wall might lead to the ureter rupture, and pus and the ureteral calculus entered the abdominal cavity, which was manifested by abdominal pain and high fever of the patient in late 2014. After pus had entered the abdominal cavity, it first caused intra-abdominal infection and then formed an intra-abdominal abscess [8]. The pus might be confined because his immune system resisted the infection. The formation of an intra-abdominal abscess is the result of the confinement of pus. The antimicrobial agents applied to him by the local general practitioner also had a therapeutic efficacy on intra-abdominal infection, as his abdominal pain and fever symptoms could be alleviated. The patient benefited from the application of antimicrobial agents and did not develop diffuse peritonitis, which might occur due to the diffusion of intra-abdominal infection. A ureteral calculus can be a cause of an intra-abdominal abscess, which has been reported in literature [5, 6]. The intra-abdominal abscess and stone of the patient might migrate to the pelvic cavity due to gravity, forming a pelvic abscess and a pelvic stone. This was confirmed by images conducted after the patient's admission in 2015. The metabolic products produced by bacteria in the abscess can penetrate into the blood [6], leading to fever because there are no endothelial cells on the wall of an abscess cavity. The patient was diagnosed with a pelvic abscess after he had admitted in 2015 and subsequently completed aspiration treatment. The follow-up MRI and B-mode sonograph were examined 3 days later after the abscess was aspirated, but CT scan was not conducted. MR images showed mixed signals existing at the original location of the abscess, and B-mode sonograph images showed high echoic shadows. The hyperechoic shadows at the original location of the abscess could not rule out the influence of some fluid drug mixture, which was injected into the pus cavity after aspiration. It was not possible to diagnose a pelvic stone at that time since it could not be denied whether the hyperechoic shadows were caused by the fluid of drug mixture. MR images also failed to diagnose the pelvic stone because it is not the modality of choice in the detection of stones, whereas the diagnosis of lithiasis usually relies on CT scan [9, 10]. The fluid in the abscess cavity might have disappeared gradually after inflammation subsides by taking antibiotics, leaving one stone in the abscess cavity. This is consistent with the images that showed a pelvic stone at the original location of the pelvic abscess on CT images in October 2023. We followed up with the patient for 6 months and found no symptoms related to his abscess after he had stopped using antibiotics in 2015. We have been unable to contact him for over 7 years, which has led to our lack of understanding of the patient's condition in recent years. Fortunately, we saw the patient recently, and he underwent a CT scan, which led to the diagnosis of a pelvic stone and confirmed that a pelvic abscess is related to the ureteral calculus. That is also why we report this case now.

The occurrence of stones in uncommon places may be casual, such as a retroperitoneal stone caused by a kidney calculus falling down during a surgical procedure [9]. The formation of an intra-abdominal abscess may also be caused by iatrogenic factors. There has been a report in the literature of a gallbladder stone falling into the abdominal cavity, causing an intra-abdominal abscess, and it also caused an intra-abdominal stone [10]. As the causes of them were clear that occurred in surgical procedures, so their etiology is different from this case. The high fever and abdominal pain of the patient at late 2014 are suspected to be caused by pus and a ureteral calculus discharged into the abdominal cavity. It demonstrates the pathogenesis of an untreated ureteral calculus causing a pelvic stone, which included a series of changes, first hydronephrosis, then infections of the ureter and renal pelvis, necrosis of the ureteral wall, an intra-abdominal abscess and stone, a pelvic abscess, and a pelvic stone at last.

The composition of urinary calculi includes oxalate calculus, uric acid calculus, phosphate calculus, etc. The calculus composed of phosphates may be infectious [11]. About 15% and 25% of phosphate calculi are infectious, which was not manifested in this case [12]. The ureteral or renal pelvis infection that first appeared in this patient may be caused by bacterial translocation [13]. The bacteria of the translocation mostly originate from the intestine, so most flora of the bacterial infection is a mixture of aerobic and anaerobic bacteria [6], which is consistent with the results of the pus culture in the patient.

We believe that the pelvic stone originating from a ureteral calculus in this patient is a noninfectious calculus, as the patient's pelvic abscess has not recurred in the past 7 years. If the patient's calculi were infectious and contained bacteria, his pelvic abscess would undoubtedly recur because antibiotics are difficult to enter the interior of the infectious calculus [14]. There is still a risk of the pelvic abscess recurring at the location of the stone because some ureteral calculi are infectious, as the pelvic stone might originate from an infectious ureteral calculus. Therefore, a patient suffering from a pelvic stone must be followed up rigorously, and it should be treated to prevent the recurrence of a pelvic abscess.

The complete obstruction of the ureter caused by a ureteral calculus should be actively treated to prevent the development of purulent nephropathy [15], which is confirmed once again by this case. If the patient's ureteral calculi were treated in time, it would not develop a pelvic abscess and a pelvic stone. In recent years, in addition to conventional drug and surgical treatments, there are many minimally invasive new methods for the treatment of ureteral and renal calculi [3, 16, 17]. These methods can be employed for patient treatment suffering from urinary calculi based on their physical conditions, which can reduce body injury. The risk of local infection can be reduced if the calculus is cured, thereby reducing the incidence of an intra-abdominal abscess as mentioned in literature or a pelvic abscess as in this case.

Pelvic stones are mostly located within the pelvic organs, which are the bladder and urinary tract [18] in the urinary system, the rectum [19] in the digestive system, and the ovaries [20], fallopian tubes [21], uterus [22], and vagina [23] in the female genital system. Male seminal vesicles can also develop stones [24] in addition to prostatic calculi [25]. There have been case reports of a stone outside the pelvic organs [26]. Therefore, when seeing images of stones in the pelvic cavity, one should first consider whether they are located within the pelvic organs or not.

There is currently no report of a pelvic stone caused by a ureteral calculus. The high-density shadows found on pelvic CT images may be tissue calcification [27], but the high-density shadows in this case are believed to be a pelvic stone caused by a ureteral calculus, based on his medical history. Therefore, this case can provide a new insight into medical literature, as a pelvic stone can originate from a ureteral calculus. A pelvic stone can locate outside pelvic organs, such as the bladder, distal urinary tract, rectum, or genital system in men and women.

The missed follow-up for 7 years had affected the observation of some characteristics of the stone. The stone might move in the patient's pelvic cavity due to it not being within a pelvic organ, but there was a gap of 7 years for not following up, so we did not know whether it moved or not, which we would never know because he had passed away.

This case illustrates the importance of follow-up in patient management, and an untreated ureteral calculus can cause complete obstruction of the ureter, leading to serious complications such as an intra-abdominal abscess, a pelvic abscess, and unrecoverable kidney atrophy, from which we have learned a lot. Therefore, urologists must be aware of the complications and pay attention to the treatment of a ureteral calculus that may cause complete obstruction to prevent serious infections and other complications.

This case also once again illustrates the importance of effective communication and interaction with patients. For this patient, clinicians should explain the serious complications of a not effectively treated ureteral calculus to him so that the patient can accept the doctor's advice. It is important to ensure that sufficient communication has been conducted before making decisions for patients in clinical practice.

## 4. Conclusion

Ureteral and renal calculi are common clinical diseases in urology. An untreated ureteral calculus can lead to serious complications of infection, such as an intra-abdominal abscess in the literature and a pelvic abscess as in this case. Long-term high pressure in the renal pelvis caused by hydronephrosis can lead to renal atrophy, which is difficult to recover. Therefore, the follow-up of patients with an untreated ureteral calculus should be highlighted in clinical practice to prevent serious complications. The infection should be considered when abdominal pain and fever occur, which is a complication of a ureteral calculus. Prompt diagnosis and treatment are required after complications occur. Minimally invasive treatment should be considered first once an abscess occurs as it causes minimal injury to the body, and antibiotics administration should be followed later. It is necessary to pay attention to their renal function in patients with a ureteral calculus because renal function can be damaged in them, which is not shown in this patient. It is very important to communicate fully with patients before making treatment decisions for them in clinical practice. We have learned a lot from the case.

There are currently no reports of pelvic abscesses and pelvic stones originating from ureteral calculi. The possibility of a pelvic stone should be considered when hyperechoic shadows in B-mode ultrasound or high-density shadows in CT images are found in pelvic examinations.

## Data Availability

The data and images used are available from the first author (corresponding author) upon reasonable request.
